# Evaluation of Dynamic Disulphide/Thiol Homeostasis in Silica Exposed Workers

**DOI:** 10.4274/balkanmedj.2015.1632

**Published:** 2017-03-28

**Authors:** Meşide Gündüzöz, Ceylan Bal, Murat Büyükşekerci, Salim Neşelioğlu, Türkan Nadir Öziş, Servet İritaş, Halil Kara, Özcan Erel

**Affiliations:** 1 Clinic of Family Medicine, Ankara Occupational Diseases Hospital, Ankara, Turkey; 2 Department of Biochemistry, Yıldırım Beyazıt University School of Medicine, Ankara, Turkey; 3 Clinic of Pharmacology, Ankara Occupational Diseases Hospital, Ankara, Turkey; 4 Clinic of Chest Diseases, Ankara Occupational Diseases Hospital, Ankara, Turkey; 5 Department of Toxicology, The Council of Forensic Medicine, Ankara, Turkey; 6 Department of Pharmacology, Yıldırım Beyazıt University School of Medicine, Ankara, Turkey

**Keywords:** Disulphide, thiol, oxidative stress, silica, exposure

## Abstract

**Background:**

Oxidative stress is implicated as one of the main molecular mechanism underlying silicosis.

**Aims:**

In this study, our aim was to asses the redox status in occupationally silica-exposed workers, by evaluating the dynamic thiol-disulphide homeostasis.

**Study Design:**

Case-control study.

**Methods:**

Thirty-six male workers occupationally exposed to silica particles and 30 healthy volunteers, working as office workers were included to the study. Posteroanterior chest radiographs and pulmonary function tests of both groups were evaluated. Also serum thiol disulphide levels were measured using the spectrophotometric method described by Erel and Neşelioğlu.

**Results:**

Among the 36 workers that underwent pulmonary function tests 6 (17%) had obstructive, 7 (19%) had restrictive, 6 (17%) had obstructive and restrictive signs whereas 17 (47%) had no signs. The mean PFTs results of silica-exposed workers were significantly lower than control subjects. The serum disulphide levels of silica-exposed workers were significantly higher than control subjects (23.84±5.89 μmol/L and 21.18±3.44 μmol/L, respectively p=0.02).

**Conclusion:**

The serum disulphide levels, a biomarker of oxidative stress, are found to be higher in silica-exposed workers.

Silicosis is an occupational pulmonary disease and exposure to crystalline silica via airways is the major aetiologic factor. The most common form is chronic silicosis that occurs following exposure to relatively low silica concentrations for over 10 years. Following exposure to exceedingly high silica concentrations, acute silicosis may develop in weeks or months, whereas accelerated silicosis may develop after 5–10 years exposure to higher silica levels (1).

Occupational exposure to crystalline silica is associated with many occupations and industries: boiler operators, stationary engineers, blockmasons, brickmasons, sandblasting, stone-cutters, crushing, grinding, polishing-machine setters, operators, and tenders. Apart from silicosis, a number of diseases, such as rheumatoid arthritis, chronic renal disease, systemic sclerosis, lupus and lung cancer have been associated with silica exposure (2).

Although silicosis has been studied extensively by many clinical and basic scientists, little is known about the cellular and molecular mechanisms underlying the disease. However, the initiating step seems to be the severe inflammation following exposure to silica particles (3). Alveolar macrophages ingest the inhaled silica particles and this leads to cell death and release of intracellular silica later taken up by other macrophages. The inflammatory process perpetuates as a result of this recurring cycle of macrophage phagocytosis and cell death (4). Macrophages that are either damaged or activated by silica particles release cytotoxic oxidants, proteases, inflammatory cytokines such as interleukin 1 (IL-1), tumour necrosis factor-α (TNF-α), and arachidonic acid metabolites. The inflammatory cells are provoked by these mediators and they recruit into the alveolar wall where they initiate alvolitis, which causes cellular damage and destroys the cellular matrix (5). Deposition of excessive extracellular matrix protein in the interstitial tissue and in the basal membrane is the main aspect of fibrosis. Oxidative stress is implicated as one of the primary mechanisms underlying fibrosis in many different organs, including the lungs (6). Crystalline silica promotes the generation of reactive oxygen species (ROS), which are directly involved in the inflammation stage of fibrosis and play an important role in the development of silicosis. ROS injure the tissue and activate the alveolar macrophages, which later go to apoptosis. ROS also enhance the synthesis of inflammatory cytokines (7). Silica-induced oxidative stress activates some specific transcription factors, such as nuclear factor kappa-light-chain-enhancer of activated B cells and activator protein 1, which results in an increase in cytokine expression (3). The generation of ROS by silica exposure has been observed in experimental studies. For example, using electron spin resonance spin-trapping techniques, it has been detected that free radicals formed on the surface of silica and ROS, including hydroxyl radical, singlet oxygen, superoxide anion, hydrogen peroxide, have been generated in cell-free environments (8). Thiols are composed of a hydrogen and sulphur atom connected to a carbon atom. The plasma thiol pool is mainly formed by protein thiols, albumin thiols and other low-molecular-weight thiols such as glutathione (GSH), cysteine, and homocysteine (9). Thiols may go through oxidation by oxidants thus covalent disulphide bonds are formed. Oxidative stress induces the formation of mixed disulphide through the oxidation of cysteine residues. These disulphide bonds can later again be reduced to thiol groups. In this way the dynamic thiol- disulphide homeostasis is sustained (10). Determination of thiol/disulphide homeostasis can provide precious information on the oxidative status of the biologic systems during any external or internal oxidative assault.

In this study, our aim was to assess the oxidative status in occupationally silica-exposed workers, by evaluating the dynamic thiol- disulphide homeostasis.

## MATERIALS AND METHODS

### Study population

Thirty-six male workers occupationally exposed to silica were included in the study. Among these 36 employees 19 were ceramic workers, 5 were denim sandblasters, 7 were welders and 5 were dental technicians. The control group included 30 healthy office workers who had no occupational exposure to silica. Mean working period of workers was 15.2±8.2 years.

Those who had one of the following criteria either in the study group or the control group were excluded from the study:

a) the presence of any acute disease or chronic disease such as - cardiovascular diseases, cerebrovascular disease, diabetes, rheumatoid arthritis, chronic kidney disease malignancy, neurodegenerative disease; b) taking any antioxidants, such as N-acetyl cysteine, lipoic acid, vitamins, or herbal supplements; c) tobacco users.

The study was approved by the local ethical committee and informed consents were taken from all participants.

### Chest radiographs and spirometry measurement

Postero-anterior (PA) chest radiographs were taken in the radiology clinic of our hospital. A short exposure time with high voltage technique was used. PA chest X-rays were evaluated by a B-reader chest disease specialist. Radiographic abnormalities of the pneumoconioses were classified into four profusion categories (0-3), regarding the concentration of small opacities in affected zones of the lung. Also 12 subcategories were determined as 0 (0/-; 0/0; 0/1),1 (1/0; 1/1; 1/2), 2 (2/1, 2/2, 2/3) or 3 (3/2; 3/3; 3/+), according to the International Labour Office (ILO) classification (11).

A standard spirometry measurement was made with a dry-seal-spirometer (Zan 100; nSpire Health Inc, Oberthulba, Germany). Pulmonary function tests were interpreted in accordance with the American Thoracic Society standards (12). Pulmonary function data included forced vital capacity (FVC) per cent pred, forced expiratory volume in a second (FEV_1_) per cent pred, FEV_1_/FVC actual, value of 25-75 per cent maximum expiratory flow (MEF_25-75_) per cent pred. A restrictive pattern was defined as an FVC <80% predicted and a normal or high FEV_1_/FVC ratio. An obstructive pattern was defined as an FEV_1_ <80% predicted and an FEV_1_/FVC ratio <0.75.

### Collection of biological samples and of measurement parameters

The blood samples obtained from workers at the end of the last working day of the week were stored at -80 °C in 16x100 mm red-capped tubes that contain no gel (BD Vacuteiner; Franklin Lakes, New Jersey) until analysis date, after having been centrifugated at 1500 g for 10 min. The spectrophotometric method defined by Erel and Neselioglu (13) was utilized to assess the serum disulphide/thiol homeostasis. Briefly; dynamic disulphide bonds (-S-S-) in the sample were reduced, as they could form free functional thiols (-SH) by NaBH4 (sodiumborohydrate). Formaldehyde was used to consume and remove the remaining reductant NaBH4 completely. The extra reduction of the 5,5’-dithiobis-2-nitrobenzoic acid (DTNB) and further reduction of the formed disulphide bonds, which are produced after DTNB, reaction is thus prevented. All thiol groups, reduced and native thiol groups, were identified at the end of a reaction by (DTNB). The concentration of dynamic disulphide was determined by the formula:

[dynamic disulphide] = [total thiol] - [native thiol]/ 2

Disulphide/total thiol per cent ratio (SS/SH+SS), disulphide/native thiol per cent ratio (SS/SH), and native thiol/total thiol percent ratios (SH/SH+SS) were calculated by using the concentrations of disulphide, native thiol and total thiol, which were previously determined.

White blood cell (WBC) numbers were determined by Beckman Coulter LH780 (Beckman Coulter, Brea, California). Erythrocyte sedimentation rate (ESR) was measured on test 1 TH (Alifax SPA, Padova, Italy). For both tests, whole blood was collected in EDTA-containing tubes (BDVacuteiner; Franklin Lakes, New Jersey) and analysed on the same day.

### Statistical analyses

Statistical Package for the Social Sciences (SPSS Inc, Chicago, IL, USA) package program (Version 18.0) was used for the statistical analysis of data. The Shapiro-Wilk test was utilized to make the coherence to normal distribution analysis. In the case of normally distributed data, values were presented as mean ± standard deviation, otherwise they were presented as median (minimum-maximum). Student’s t test and Mann-Whitney U test were used to examine the existence of a statistically significant difference among the groups in terms of continuous variable tests for parametric variables and for non-parametric variables, respectively. The existence of a statistically significant difference among the groups was determined by Kruskall-Wallis test with Bonferrroni correction when parameters were non-normally distributed, or with one-way analysis of variance post hoc Bonferroni test, when parameters were normally distrubuted. All results were considered statistically significant for p<0.05. Area under curve (AUC), specificity and sensitivity values were calculated with receiver operating characteristic (ROC) analysis. Post-hoc power of the study was calculated with PASS Sample Size Software (PASS 14, NCSS, Utah, USA).

## RESULTS

The study comprised 36 silica-exposed workers and 30 control subjects. All the participants underwent pulmonary function tests (PFTs). Among the 36 workers that underwent PFTs, 6 (17%) had obstructive, 7 (19%) had restrictive, 6 (17%) had obstructive and restrictive signs, and 17 (47%) had no signs. All the control subjects had normal PFT results. The results of the PFTs: FVC per cent predicted (FVC % pred.), FEV_1_ per cent predicted (FEV_1_ % pred.), FEV_1_/FVC actual and MEF_25-75_ per cent predicted (MEF_25-75_% pred.) were significantly lower in silica-exposed workers when compared with control subjects (p<0.001) (Table 1). The profusion categories of the workers were 7 (19%) with category 0, 8 (22%) with category 1, 14 (39%) with category 2 and 7 (19%) with category 3, according to the ILO classification. The PFTs were impaired in all profusion categories except for category 0 (Table 2). The serum disulphide levels of silica-exposed workers were significantly higher than control subjects (23.84±5.89 and 21.18±3.44 respectively p=0.02) (Table 1). The serum disulphide levels of silicosis patients in ILO profusion categories 0 and 2 were found significantly higher than control subjects (Table 2). Also, the disulphide to native thiol and disulphide to total thiol % ratios were higher in workers than control subjects but this was not statistically significant (Table 1).

Disulphide had 67% sensitivity and 70% specificity for 22.15 μmol/L cut-off value. According to ROC analysis the AUC for disulphide was 0.667. Post hoc power of the study was 0.63. There was no significant difference between control group (7.08±1.69x10^3^/µL) and patient group (6.92±1.68x10^3^/µL) in terms of mean WBC count (p=0.707). Patient group’s median ESR values were significantly higher than control group (p=0.013). Median (minimum-maximum) ESR values of the control and patient group were 4 (1-18) mm/h and 6.5 (1-38) mm/h, respectively.

## DISCUSSION

Pulmonary tissues are constantly exposed to ROS originating from a variety of exogenous or endogenous sources. Lungs’ own unique defence mechanisms to combat the unpredictable exposure to ROS involves antioxidant enzymes and non-enzymatic defences. As a delicate balance exists between ROS generation and antioxidant systems, an oxidative stress is induced, resulting in cell injury when the balance is overwhelmed by either an excessive production of ROS or loss of antioxidant defences (14).

In this study, the silica-exposed workers underwent pulmonary function tests; 17% had obstructive signs, 19% had restrictive, 17% had obstructive and restrictive signs and 47% had no signs. In a recent study, which described the epidemiological and clinical characteristics of occupational silicosis, it was shown that PFTs in simple silicosis cases (n = 42) revealed a very moderately restrictive pattern, while the four complicated silicosis cases showed a more restrictive spirometric profile (15).

Ergün et al. (16) explored the rate of pneumoconiosis in dental technicians and evaluated the risk factors. They reported that of the patients with pneumoconiosis (n=87) 28.7% had obstructive PFT signs, 9.2% restrictive PFT signs and 62.1% had normal PFT signs.

Silicosis is an occupational disease that currently has no effective treatment. It is an incurable but preventable disease. Pulmonary injury develops as a result of inhalation of crystalline silica nanoparticles and this results with progressive lung fibrosis (17). Oxidative stress is thought to be one of the main mechanisms in the development of fibrosis induced by silica. In silicosis, a complex sequence of reactions occurs in the perpetuation of the phagocytic and inflammatory process and continued generation of ROS. The combined effect of free radical formation, repeated phagocytosis and augmented inflammatory processes may lead to oxidative stress, which leads to deleterious effects in the pulmonary tissues if the antioxidant defence systems are insufficient (14,18). Zhang et al. (19) found a dose and time-dependent relationship between the involvement of ROS formation and oxidative stress in silica-induced cytotoxicity and genotoxicity in cultured rat alveolar macrophages. They also observed that the antioxidant enzymes superoxide dismutase and catalase inhibited silica-induced oxidative stress in alveolar macrophages. It has been demonstrated that administration of silica in lung tissue or cells induced ROS formation and up-regulation of antioxidant enzymes in experimental studies (14,20).

High oxidative stress as determined by increased lipid peroxidation products, such as malondialdehyde (MDA) levels and decreased GSH content, together with pulmonary dysfunctions induced by crystalline silica exposures were shown in brick kiln workers (21). Villarini et al. (22) found increased frequency of micronucleus a biomarker of oxidatively damaged DNA in tunnel construction workers exposed to silica as compared to controls (22). Wallaert et al. (23) reported that a relationship existed between the level of oxidants produced by pulmonary phagocytes and lung damage and severity of pneumoconiosis. They also observed that spontaneous superoxide anion generation was three- to fourfold higher than controls in patients with single pneumoconiosis. Palabiyik et al. (24) investigated the antioxidant enzyme activity in denim sandblasting workers and concluded that the superoxide dismutase activity was enhanced and this was considered as an early indicator of the effects of silica and could be interpreted as a compensatory mechanism in response to the increased ROS generation caused by silicosis. Orman et al. (25) reported that increased MDA levels and reduced GSH levels in red cells of cement plant workers were associated with silicosis, providing an oxidative link. In this study, we found that the serum disulphide levels of silica-exposed workers were significantly higher than control subjects. Also, the disulphide to native thiol and disulphide to total thiol % ratios of silica-exposed workers were higher in silicosis patients than control subjects, but this was not statistically significant. The direction of many cellular processes depends on the ‘redox state’, which is a term that has been used to describe the ratio of the interconvertible oxidized and reduced form of a specific redox couple, such as thiol/disulphide. The redox state of the thiol/disulphide couple can serve as an important indicator of oxidative stress (26). GSH comprises the main intracellular thiol pool and is among the most important antioxidants in cells. It is utilized in enzymatic reactions and to eliminate peroxides and in non-enzymatic reactions to maintain the antioxidants such as α-tocopherol and ascorbic acid in their reduced and functional forms (27). The GSH-dependent detoxification of ROS is achieved through two general mechanisms: i) reacting with ROS directly or spontaneously; ii) decomposition of ROS catalyzed by GSH peroxidase. GSH disulphide (also known as oxidized GSH) is produced at the end of these reactions (28). Thiols, the functional group of GSH, due to its chemical reactivity can undergo a wide range of modifications, which involve reversible oxidation of the thiol to disulphide, including intra- and intermolecular disulphides between polypeptides and GSH-polypeptide. The reversibility of these oxidation reactions allows thiol groups to act as versatile transducing elements in a variety of low molecular weight mass metabolites and proteins. Antioxidant defence is recognized to be modulated at a certain stage by thiol/disulphide exchange mechanisms (29). Thiol groups are oxidized to disulphides during oxidative stress, thus thiol/disulphide ratio offers a simple and convenient expression of cellular oxidative stress (30). Recently, thiol disulphide homeostasis has been investigated in many disorders, such as acute myocardial infarction, type 1 diabetes and acute ischaemic stroke (31,32,33).

It was suggested that the relatively higher disulphide levels measured in silica-exposed workers are a result of oxidative stress induced by silica particles. Also, we observed that the serum disulphide levels of the workers in category 0 and 2, but not the patients in category 1 and 3, were significantly higher than the control group. It can be suggested that in the severe form of the illness that might correspond to the chronic stage, the antioxidant system of the body partially overwhelms the oxidative state. In severe forms of the disease, a worsening of the pulmonary functions that may result in chronic hypoxia and oxidative stress may be expected, but this is not the case according to our findings. The pulmonary function tests worsen with advancing categories but the disulphide levels do not show an increase parallel to this worsening.

As a result, oxidative stress plays an important role in the pathogenesis of silicosis. As previously reported in a number of experimental and clinical studies, the biomarkers of oxidative stress seem to be higher in silicosis. We revealed that disulphide levels, a biomarker of oxidative stress, were significantly higher in occupationally silica-exposed workers. In the diagnosis of silicosis, this method will enable the clinician to determine the oxidative status of patients in a practical, easy, inexpensive and fully automated way. As thiol/disulphide homeostasis is disturbed in silicosis patients, according to the further results of researches the thiol-donating agents and antioxidants might be recommended as therapeutic interventions.

Our study has some limitations. First, air samples surrounding the breathing zones of workers could not be monitored and airborne silica concentrations were not measured. A second limitation is that other oxidative stress markers, such as antioxidant enzymes (catalase, superoxide dismutase, GSH peroxidase) and lipid peroxidation products (MDA, isoprostane) were not determined.

## Figures and Tables

**Table 1:**
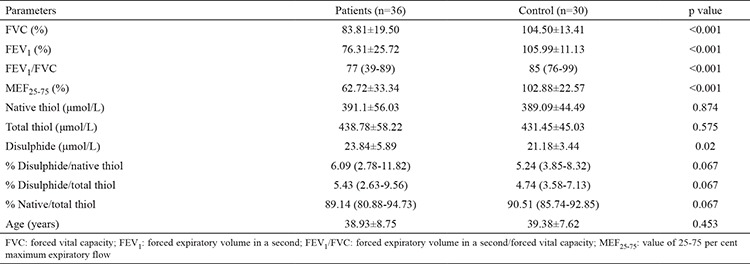
Pulmonary function test results and thiol disulphide levels of Silica-exposed workers and control subjects

**Table 2:**
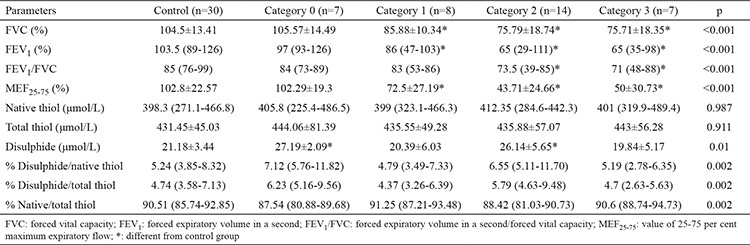
Pulmonary function tests results and native thiol, total thiol, disulphide levels of control subjects and silicosis patients according to the profusion cathegories
